# The Cotton BEL1-Like Transcription Factor GhBLH7-D06 Negatively Regulates the Defense Response against *Verticillium dahliae*

**DOI:** 10.3390/ijms21197126

**Published:** 2020-09-27

**Authors:** Qiang Ma, Nuohan Wang, Liang Ma, Jianhua Lu, Hantao Wang, Congcong Wang, Shuxun Yu, Hengling Wei

**Affiliations:** 1 State Key Laboratory of Cotton Biology, Institute of Cotton Research of Chinese Academy of Agricultural Science, Anyang 455000, China; 20200066@ayit.edu.cn (Q.M.); maliang3417@163.com (L.M.); lujianhua@caas.cn (J.L.); wanghantao@caas.cn (H.W.); wangcongc1992@163.com (C.W.); 2 College of Biology and Food Engineering, Anyang Institute of Technology, Anyang 455000, China; 20190031@ayit.edu.cn

**Keywords:** cotton, GhBLH7-D06, lignin biosynthesis, JA signaling pathway, GhOFP3-D13, Verticillium wilt

## Abstract

Verticillium wilt will seriously affect cotton yield and fiber quality. BEL1-Like transcription factors are involved in the regulation of secondary cell wall (SCW) formation, especially the biosynthesis of lignin that also plays a key role in cotton disease resistance. However, there is no report on the role of BEL1-Like transcription factor in the regulation of plant biological stress. In this study, tissue expression pattern analysis showed that a BEL1-Like transcription factor GhBLH7-D06 was predominantly expressed in vascular tissues and the SCW thickening stage of fiber development, while its expression could also respond to *Verticillium dahliae* infection and the phytohormone MeJA treatment, which indicated that GhBLH7-D06 might be involved in the defense response of Verticillium wilt. Using virus-induced gene silencing (VIGS) technology, we found silencing the expression of *GhBLH7-D06* could enhance the resistance of cotton plants to Verticillium wilt, and the acquisition of resistance might be mainly due to the significant overexpression of genes related to lignin biosynthesis and JA signaling pathway, which also proves that GhBLH7-D06 negatively regulates the resistance of cotton to Verticillium wilt. Based on the results of yeast two-hybrid (Y2H) library screening and confirmation by bimolecular fluorescence complementary (BiFC) experiment, we found an Ovate Family Protein (OFP) transcription factor GhOFP3-D13 which was also a negative regulator of cotton Verticillium wilt resistance could that interacts with GhBLH7-D06. Furthermore, the dual-luciferase reporter assay and yeast one-hybrid (Y1H) experiment indicated that GhBLH7-D06 could target binding to the promoter region of *GhPAL-A06* to suppress its expression and eventually lead to the inhibition of lignin biosynthesis. In general, the GhBLH7-D06/GhOFP3-D13 complex can negatively regulate resistance to Verticillium wilt of cotton by inhibiting lignin biosynthesis and JA signaling pathway.

## 1. Introduction

Cotton (*Gossypium hirsutum* L.) is a globally cultivated economic crop and is a major source of natural fiber and oil [1]. Cotton production has decreased recently due to a series of environmental constraints, especially various biotic and abiotic stresses such as Verticillium wilt and drought, while the area that cotton is cultivated has declined due to increased cost of cultivation and reduction in available farmland [2,3]. Verticillium wilt is a vascular disease caused by the soil-borne fungal pathogen *V. dahliae*, which infects over 200 dicotyledonous species including cotton. Besides, it can occur throughout the whole lifespan of cotton and will cause a dramatic loss of cotton yield and a serious effect on fiber quality [4,5,6]. Verticillium wilt infects cotton by penetrating the roots and then spreads across the root cortex to invade the xylem vessels where it forms the hyphae and conidia responsible for the colonization of vascular tissues and functional impairment. This results in several symptoms, including wilting, discoloration, necrosis, defoliation and finally death [7,8,9,10].

A promising and environmentally friendly strategy to reduce the above losses is to enhance the immune system of plants via genetic engineering, and this is based on insight into the molecular mechanisms of interactions between plant and pathogen [11]. Plants use a series of defense mechanisms to protect themselves against pathogen attack through a complex perception, transduction, and exchange of signals [12]. Plant disease resistance response is a signal transduction network regulated by a series of complex signal molecules and transcription regulators. Phytohormones, such as jasmonic acid (JA), salicylic acid (SA), and ethylene (ET), play an important role in this process [13]. Studies have shown that JA is involved in the plant’s defense response to necrotrophic pathogens and plays pivotal roles in cotton’s resistance to *V. dahliae* [14,15,16]. For example, a Jasmonate-ZIM-domain (JAZ) protein, GhJAZ2, which was induced by methyl jasmonate (MeJA) and inoculation of *V. dahliae*, can interact with GhbHLH171, inhibit its transcriptional activity, and, as a result, restrain the JA-mediated defense response [17]. The WRKY transcription factor *GbWRKY1*, the expression of which is induced rapidly by MeJA and infection of *V. dahliae*, is a critical regulator mediating the plant defense-to-development transition during *V. dahliae* infection by activating *JAZ1* expression [18]. Beyond that, a member of the PINTOX homeobox proteins, HDTF1, and a HD-ZIP I homeobox transcription factor, GhHB12, both negatively regulate cotton resistance to *V. dahliae* by suppressing JA-response genes [19,20]. The calcium-dependent protein kinase GhCPK33 from upland cotton (*Gossypium hirsutum*) is also a negative regulator of resistance to *V. dahliae* that directly manipulates JA biosynthesis [21]. Suppressor of BIR1–1 (SOBIR1) is a receptor-like kinase (RLK) initially identified as a suppressor of BIR1 (BAK1-interacting receptor-like kinase 1) and plays a positive role in *Arabidopsis* immunity [22]. SA is one of the key defense-related hormones in activating the defense responses against biotrophic and hemi-biotrophic pathogens [23]. Knock-down of *GbTSA1* (Tryptophan Synthase α) and *GbTSB1* (Tryptophan Synthase β) induces a spontaneous cell death phenotype in a SA-dependent manner and enhanced resistance to *V. dahliae* in cotton plants [24]. Gong et al. reported that the SA signaling pathway mediated by GaRPL18 enhances the cotton resistance to Verticillium wilt [25]. Moreover, phytohormone crosstalk networks play important roles during plant abiotic and biotic stress responses [26]. For example, the transcription factor WRKY70, a node of convergence for SA- and JA-mediated signals for plant defense, has been shown to also be a negative regulator during the beginning of ABA-controlled stomatal closure [27,28]. GhATAF1, a NAC transcription factor, confers abiotic and biotic stress responses by regulating phytohormonal signaling networks [29].

In addition to phytohormones, mounting evidence confirms that Verticillium wilt resistance is directly associated with lignin accumulation in plants [30,31,32]. In *Arabidopsis*, plants inoculated with *Verticillium longisporum* promote novel vascular formation and lignin synthesis [33]. The *Ve*-mediated resistance response of tomato to *V. dahliae* also involves lignin and *PAL* gene expression [34]. In cotton, a series of lignin synthesis enzymes is upregulated after the cotton plants inoculation with *V. dahliae*, resulting in lignin accumulation [8,13,35,36]. For example, *GhLAC15* enhanced Verticillium wilt resistance via increasing defense-induced lignification and arabinose and xylose accumulation in the cell wall of *G. hirsutum* [37]. GhLac1 is a key regulator in a complex and interacting pathway at the interfaces of phenylpropanoid, JA, and SA metabolism [38]. The expression of *GbERF1-like* was upregulated by *V. dahliae* infection and by ET and MeJA treatments. GbERF1-like acts as a positive regulator in the resistance of plants to *V. dahliae* via directly activating the expression of genes involved in lignin synthesis, such as *GhHCT1* [39]. These reports document that lignin plays important roles in plant defense. Interestingly, the three-amino-acid-loop-extension (TALE) superclass of homeobox transcription factors, which comprise the KNOTTED-like homeodomain (KNOX) and BEL1-Like homeodomain (BLH/BELL) two family proteins, have been reported to play an important role in the regulation of SCW formation, and especially the synthesis and metabolism of lignin. For example, the BEL1-Like transcription factor BLH6 and the KNOX protein KNAT7 interact and regulate SCW formation via repression of REVOLUTA. The BELL-type homeobox gene *OsSH5* induces seed shattering by enhancing abscission-zone development and inhibiting lignin biosynthesis in rice. The KNOX protein OSH15 induces grain shattering by repressing lignin biosynthesis genes [40,41,42]. Nevertheless, few reports have reported that TALE transcription factors are involved in the regulation of fungal pathogen response. Besides that, a rice BEL-Like homeodomain transcriptional factor *OsBIHD1*, which was upregulated rapidly during the incompatible interaction between *Magnaporthe grisea* and a resistant genotype rice, is associated with resistance response [43]. The cotton KNOX protein GhBP1 could inhibit the expression of *GhBOP1* induced by *V. dahliae* infection, and the induced expression of *GhBOP1* could enhance the resistance to *V. dahliae* by enhancing the activation activity of its interaction protein GhTGA3 and the expression of lignin synthesis-related genes [44]. Our previous study showed that many BEL1-Like family members are involved in the regulation of cotton fiber and vascular tissue SCW synthesis, and they are often functionally redundant, especially members with similar expression patterns [45]. We investigated whether cotton BEL1-Like family members participate in the resistance response to *V. dahliae*.

In the present study, we showed that GhBLH7-D06 participates in the regulation of fungal pathogen responses in cotton. The expression of *GhBLH7-D06* was dominant in vascular tissues and could be induced by MeJA treatment and response to *V. dahliae* infection. The knockdown of *GhBLH7-D06* through VIGS in cotton increased the resistance to Verticillium wilt. Further experiments demonstrated that GhBLH7-D06 localized to the nucleus and functions as a negative regulator of cotton defense against *V. dahliae* by manipulating JA signaling pathway and lignin biosynthesis via interacting with GhOFP3-D13. Taken together, these findings shed light on the molecular mechanism of BEL1-Like homologous proteins in plant defense, and the identification of BLH7-D06/OFP3-D13 complex in this study has important guiding value for discovering and understanding the role of BEL1-Like/OFP complex module in regulating cotton lignin biosynthesis and JA signal transduction.

## 2. Results

### 2.1. Identification and Characterization of a BEL1-Like Transcription Factor GhBLH7-D06

**Figure 1:**
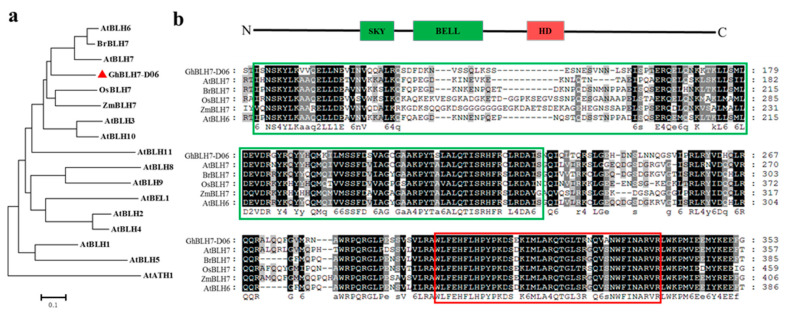
Phylogenetic analysis and sequence alignment of GhBLH7-D06 protein: (**a**) phylogenetic analysis of GhBLH7-D06 with its homologous proteins; and (**b**) multiple sequence analysis of GhBLH7-D06 with its homologous proteins AtBLH7 (AT2G16400.1), AtBLH6 (AT4G34610.1), OsBLH7 (LOC_Os12g43950.1), ZmBLH7 (GRMZM2G004641_P03), and BrBLH7 (Brara.K00337.1.p). The POX (SKY + BELL) domain sequence is in the green boxes, and the Homeobox KN domain (HD) is in the red box.

Studies have shown that many genes associated with lignin deposition in the cell wall and xylem vessels participate in cotton plant defense against *V. dahliae* [8,13]. Our previous research showed that many members of cotton BEL1-Like homeoproteins function in SCW formation in fiber and interfascicular, especially the synthesis and metabolism of lignin, which also participates in plant resistance to *V. dahliae* [45]. Thus, we were prompted to explore the cotton BEL1-Like protein GhBLH7-D06 (Gh_D06G0030) functions in plant defense and lignin synthesis. The gene *GhBLH7-D06* was cloned from upland cotton TM-1. The CDS length of the gene was 1581 bp, including four exons, encoding 526 amino acids. The amino acid sequence of the gene was used for phylogenetic tree analysis with the homologous sequence of the member of BEL1-Like family in *Arabidopsis* and GhBLH*7*-D06 homologous sequence in rice, maize, and rape (Figure 1a). At the same time, multiple sequence alignment and motif analysis also showed that GhBLH7-D06 contains the intermediate POX domain (also can be divided into the SKY and BELL motif) and the C-terminal homeobox KN domain (HD), and it is relatively conservative in various plant species (Figure 1b).

**Figure 2:**
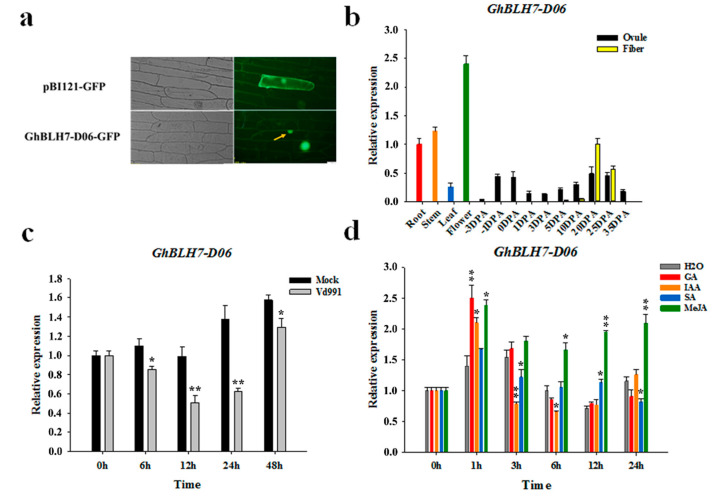
The expression patterns of *GhBLH7-D06* in cotton: (**a**) the subcellular localization of GhBLH7-D06; (**b**) the expression level of *GhBLH7-D06* in different tissues in TM-1; (**c**) the expression pattern of *GhBLH7-D06* in response to *V. dahliae* (*Vd991*) infection; and (**d**) the expression pattern of *GhBLH7-D06* responds to phytohormones (GA, IAA, SA and MeJA). Error bars represent the standard deviation of three biological replicates. Asterisks indicate statistically significant differences, as determined by Student’s *t*-test (* *p* < 0.05; ** *p* < 0.01).

The subcellular localization of a protein is closely related to their normal functions. To confirm this, the constructed transient expression fusion vector pBI121-GhBLH7-D06-GFP and control vector pBI121-GFP were transformed into *Agrobacterium tumefaciens* by the method of onion epidermal cell infection. After coculturing, the appropriate part was observed under the fluorescence microscope. The GFP-GhBLH7-D06 fusion protein preferentially accumulated in the nucleus, whereas the GFP was distributed throughout the cell (Figure 2a).

### 2.2. Expression Pattern Analysis of Cotton GhBLH7-D06

To further speculate on the biological function of GhBLH7-D06, the tissue expression pattern and induced expression pattern of *GhBLH7-D06* were identified. Primers were designed in the 3’UTR region of *GhBLH7-D06*, and the correlation analysis was carried out by qRT-PCR. Tissue expression pattern analysis showed that *GhBLH7-D06* was ubiquitously expressed in all tissues, but it was predominantly expressed in vascular tissues (root and stem), flower, and 20–25 dpa of the fiber secondary wall thickening stage (Figure 2b).

The induced expression pattern showed that the expression level of *GhBLH7-D06* decreased significantly under the infection of *V. dahliae*, indicating that it may be a negative regulator of cotton Verticillium wilt resistance (Figure 2c). MeJA, as an important phytohormone involved in the plant immune system, could quickly upregulate transcription of *GhBLH7-D06*, and *GhBLH7-D06* was also upregulated by gibberellin (GA), which may be involved in the regulation of organ growth and fiber development. Moreover, *GhBLH7-D06* transcription did not respond to the treatments of auxin (IAA) and SA (Figure 2d).

### 2.3. GhBLH7-D06 Silenced Cotton Displays Enhanced Resistance to V. dahliae Infection

**Figure 3:**
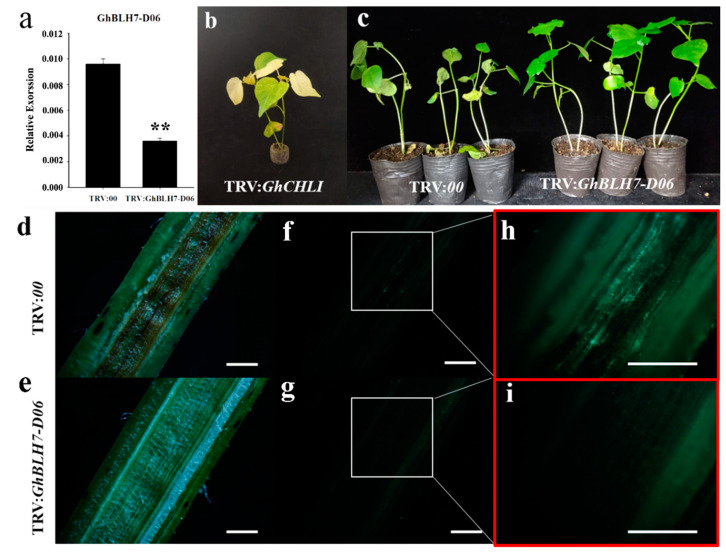
Phenotypic identification and analysis of *GhBLH7-D06* VIGS cotton: (**a**) silencing efficiency of VIGS test of *GhBLH7-D06*; (**b**) phenotype of VIGS positive control plants; (**c**) phenotype observation of *GhBLH7-D06* VIGS plants and wild type control plants after two weeks of *V. dahliae* (*Vd991-GFP*) infection; and (**d**–**i**) observation of epicotyl longitudinal sections of *GhBLH7-D06*-silenced plants and control plants, Scale bars  =  1.0 mm. Error bars represent the standard deviation of three biological replicates. Asterisks indicate statistically significant differences, as determined by Student’s *t*-test (** *p* < 0.01).

VIGS is a rapid and effective technique to further investigate the biofunction of GhBLH7-D06 in cotton resistance to Verticillium wilt, the VIGS vector TRV:*GhBLH7-D06* was transformed into *A. tumefaciens* and infected with the receptor plants TM-1 to generate the *GhBLH7-D06*-silenced plants. The expression level of *GhBLH7-D06* in control plants TRV:*00* and experimental plants TRV:*GhBLH7-D06* were identified by qRT-PCR, and the silencing efficiency was calculated during the leaves of positive control plants TRV:GhCHLI showed yellowing phenotype (Figure 3a,b). Two weeks later, we observed the phenotype of *V. dahliae* (*Vd991-GFP*) infection in the control group and experimental group with high silencing efficiency. It was found that TRV:*00* in the control group had symptoms of leaf wilting and falling off, withering and yellow, and the plant growth was slow, while TRV:*GhBLH7-D06* in the experimental group had relatively normal growth (Figure 3c). After cutting the epicotyl of the two group plants, it was found that the stem pith in the control group plants were obviously browning, but the experimental group was still normal, indicating that the infection of *V. dahliae* on TRV:*GhBLH7-D06* plants in the experimental group was weak (Figure 3d,e). Fluorescence microscope was used to observe the infection status of *V. dahliae* in the epicotyl of the plant. It was found that there were more *V. dahliae* (*Vd991-GFP*) in the control group, while the content in experimental group was less, indicating that the silencing of *GhBLH7-D06* significantly inhibited the colonization and transmission of *V. dahliae* (Figure 3f–i).

### 2.4. Silencing of GhBLH7-D06 in Cotton Activates the Biosynthesis of Lignin and JA Associated Genes

**Figure 4:**
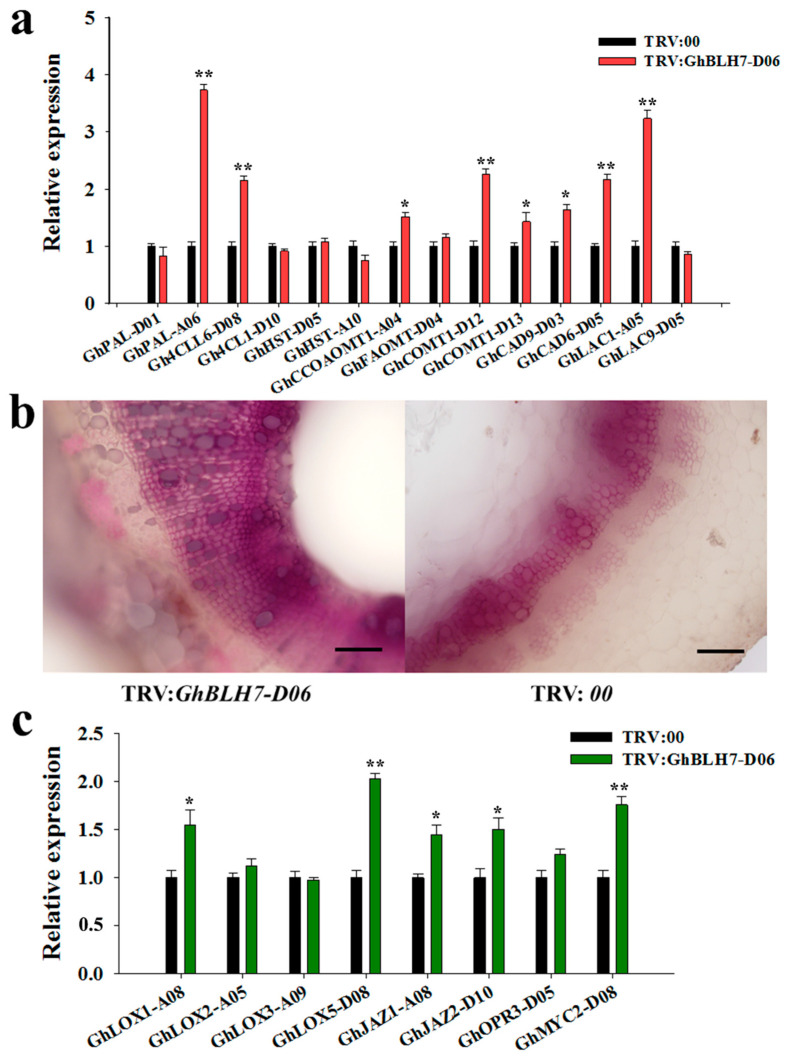
Observation and detection of lignin components in *GhBLH7-D06*-silenced plants and control plants: (**a**) expression analysis of lignin synthesis-related genes in *GhBLH7-D06*-silenced plants and control plants; (**b**) observation of lignin phloroglucinol staining sections of *GhBLH7-D06*-silenced plants and control plants (scale bars  =  0.1 mm); and (**c**) expression analysis of JA signaling pathway genes in *GhBLH7-D06*-silenced plants and control plants. Error bars represent the standard deviation of three biological replicates. Asterisks indicate statistically significant differences, as determined by Student’s *t*-test (* *p* < 0.05; ** *p* < 0.01).

Several studies have suggested that lignin metabolism confers resistance to Verticillium wilt in cotton [22,24]. To validate the participation of GhBLH7-D06 in the lignin metabolic pathway, the expression of lignin synthesis-related genes (including *GhPAL-D01*, *GhPAL-A0*6, *Gh4CLL6-D08*, *Gh4CL1-D10*, *GhHST-D05*, *GhHST-A10*, *GhCCoAOMT-A04*, *GhFAOMT-D04*, *GhCOMT1-D12*, *GhCOMT1-D13*, *GhCAD9-D03*, *GhCAD6-D05*, *GhLAC1-A05* and *GhLAC9-D05*) were examined in two groups of VIGS plants. In the *GhBLH7-D0*6-silenced cotton plants, the expression levels of *GhPAL-A06*, *Gh4CLl6-D08*, *GhCCoAOMT-A04*, *GhCOMT1-D12*, *GhCOMT1-D13*, *GhCAD9-D03*, *GhCAD6-D05* and *GhLAC1-A05* was significantly increased in the experimental group, among which the expression of *GhPAL-A06* was 2.73 times higher than that of the control group, indicating that GhBLH7-D06 was negative for lignin biosynthesis (Figure 4a).

Furthermore, to confirm the above results, the phloroglucinol-HCl (Wiesner) reaction was applied to detect the content of lignin in stems. The plants were collected 14 days after inoculation with *V. dahliae*, and hand-cut cross-sections of epicotyls were prepared for Wiesner staining. The lignin content of vascular tissue increased significantly in *GhBLH7-D06*-silenced plants (Figure 4b).

Because of the phytohormone JA play a major, predominantly antagonistic regulatory roles in cotton resistance to Verticillium wilt and it can significantly induce the expression of *GhBLH7-D06*, we also analyzed the transcript levels of JA signaling pathway genes in VIGS plants. The results show that *GhBLH7-D06*-silencing result in the upregulation of the expression levels of genes related to JA biosynthesis and signaling, such as *GhLOX1-A08*, *GhLOX2-A05*, *GhLOX3-A09*, *GhLOX5-D0*8, *GhJAZ1-A08*, *GhJAZ2-D10*, *GhOPR3-D05* and *GhMYC2-D08* (Figure 4c). These results suggest that knockdown of *GhBLH7-D06* could enhance the disease resistance response in cotton vascular tissues.

### 2.5. GhBLH7-D06 Interacts with GhOFP3-D13 in Response to V. dahliae Infection

In the previous report, we identified the transcriptional self-activation activity of several members of the BEL1-Like family, which proved that GhBLH7-D06 had no self-activation activity [44]. Therefore, we used the Y2H system to study the interaction between proteins and clarify the regulatory mechanism of GhBLH7-D06 transcription factor in the regulation of cotton SCW thickening and response to the infection of *V. dahliae*. In the Y2H library screening experiment, the yeast hybrid library of cotton stem and leaf bud mixed sample was used as the bait library to screen the GhBLH7-D06 interaction proteins. In total, 32 positive clones were obtained, including one KNOX family protein, knotted-1-like 3 (GhKNAT3-A13), and one OFP family protein, OFP3-like (GhOFP3-D13) (Table S1).

**Figure 5:**
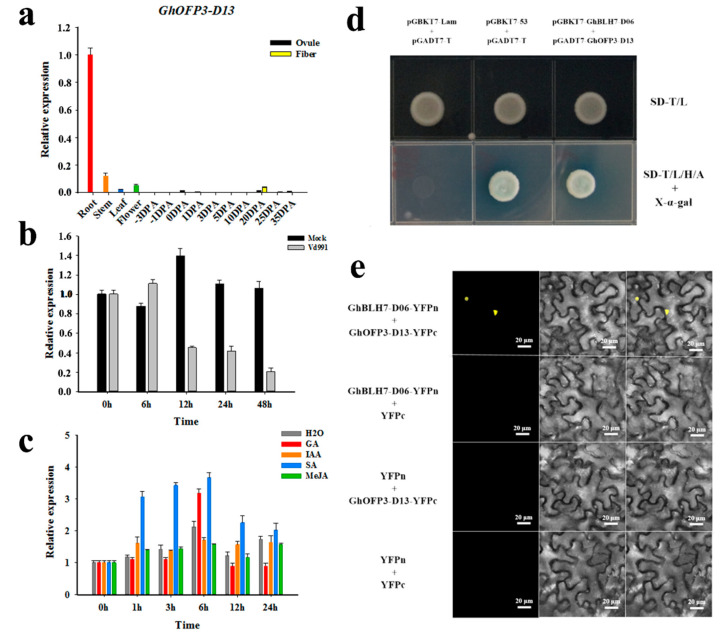
Validation of interaction between GhBLH7-D06 and GhOFP3-D13 and the expression patterns of GhOFP3-D13 in cotton: (**a**) the expression level of *GhOFP3-D13* in different tissues in TM-1; (**b**) the expression pattern of *GhOFP3-D13* in response to *V. dahliae* (*Vd991*) treatment; (**c**) the expression patterns of *GhOFP3-D13* responds to phytohormones (GA, IAA, SA and MeJA); (**d**) negative control group (pGADT7-T + pGBKT7-lam), positive control group (pGADT7-T + pGBKT7-53), and experimental group (pGADT7-GhOFP3-D13 + pGBKT7-GhBLH7-D06) grown on two-deficiency medium SD-T/L (SD-Trp/-Leu) and four-deficiency medium SD-T/L/H/A + X-α-gal (SD-Trp/-Leu/-His/-Ade + X-α-gal); and (**e**) BiFC assays for determination of GhBLH7-D06 and GhOFP3-D13 interactions in *Nicotiana benthamiana* leaf cells (scale bar = 20 μm). Error bars represent the standard deviation of three biological replicates. Asterisks indicate statistically significant differences, as determined by Student’s *t*-test.

According to the screening results of the bait protein GhBLH7-D06, we further verified and analyzed the KNOX family and OFP family members as the candidate target proteins. Finally, by analyzing the expression patterns, we selected the OFP family protein GhOFP3-D13 as the candidate capture protein for point-to-point verification. As *GhOFP3-D13* was the preponderant expression in the stem and with low expression in other tissues and organs, it can be regarded as a stem specific expression gene. In addition, the expression level of *GhOFP3-D1*3 decreased significantly under the infection of *V. dahliae*, and the expression of *GhOFP3-D13* was significantly induced by SA and could respond to GA treatment, but did not obviously respond to the IAA and MeJA treatment (Figure 5a–c). The AD expression vector pGADT7-GhOFP3-D13 was cloned and constructed. By co-transformation, pGADT7-GhOFP3-D13 + pGBKT7-GhBLH7-D06, positive control pGADT7-t + pGBKT7-53, and negative control pGADT7-t + pGBKT7-lam were transformed into Y2H gold yeast cells. The positive transformants were identified by PCR and cultured on the defective medium. The results show that all the transformed products grew well on *SD*-T/L deficient medium, and the positive control group and experimental group could grow well on *SD*-T/L/H/A + X-α-gal deficient medium (Figure 5d), indicating that GhBLH7-D06 could interact with GhOFP3-D13 to form heterodimer to jointly regulate the synthesis of SCW of cotton fibers and the response to infection of *V. dahliae*.

To further confirm the reliability of the interaction between GhOFP3-D13 and GhBLH7-D06, we constructed GhBLH7-D06-YFPn and GhOFP3-D13-YFPc, respectively, for BiFC experiment. The fusion expression vector of yellow fluorescent protein (YFP) was transiently expressed in tobacco leaves by *A. tumefaciens* injection, and the signal of YFP was observed under laser confocal microscope. The results show that GhBLH7-D06-YFPn and GhOFP3-D13-YFPc are co-expressed in tobacco leaves, which can generate yellow fluorescence signal, indicating that GhBLH7-D06 and GhOFP3-D13 can interact (Figure 5e).

### 2.6. GhBLH7-D06 Could Inhibit the Expression of GhPAL-A06

In the VIGS plants of *GhBLH7-D06*, the expression of most genes related to lignin biosynthesis was significantly increased, which indicated that GhBLH7-D06 had a significant inhibitory effect on lignin biosynthesis, and it was more likely that GhBLH7-D06 directly inhibited the expression of lignin biosynthesis genes. As a metabolizing enzyme gene upstream of lignin metabolic pathway, *GhPAL-A06* showed high differential expression multiple in *GhBLH7-D06*-silenced plants. We speculated that the expression of *GhPAL-A06* could be directly regulated by GhBLH7-D06. The dual-luciferase reporter assay and Y1H experiment were employed to validate the interaction of GhBLH7-D06 with *GhPAL-A06* promoter sequence.

**Figure 6:**
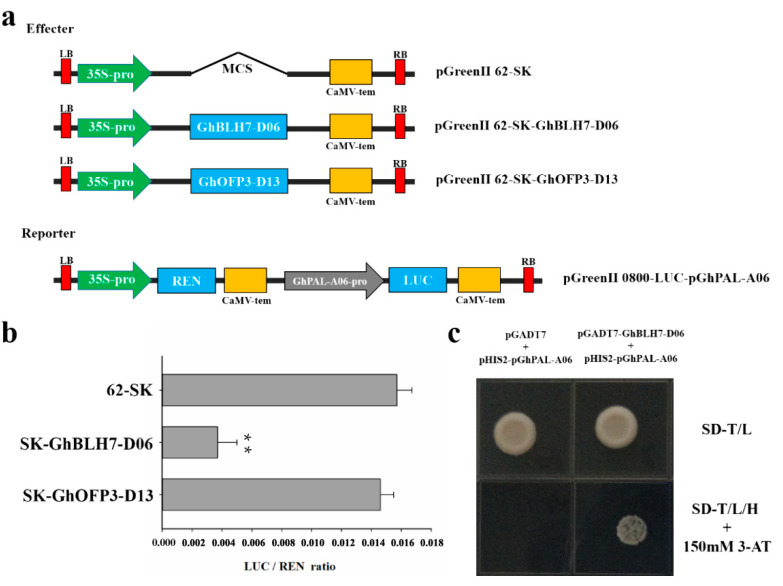
Validation the targeting regulation of GhBLH7-D06 on *GhPAL-A06*: (**a**,**b**) the targeting regulation of GhBLH7-D06 and GhOFP3-D13 on *GhPAL-A06* by dual-luciferase reporter assay system; and (**c**) validation of interaction between GhBLH7-D06 and *GhPAL-A06* promoter by Y1H. The empty vector control group (pHIS2-pGhPAL-A06 + pGADT7) and the experimental group (pHIS2-pGhPAL-A06 + pGADT7-GhBLH7-D06) were grown on two-deficient medium SD-T/L (SD-Trp/-Leu) and three-deficient medium SD-T/L/H + 150 mM 3-AT (SD-Trp/-Leu/-His + 150 mM 3-AT). Error bars represent the standard deviation of three biological replicates. Asterisks indicate statistically significant differences, as determined by Student’s *t*-test (** *p* < 0.01).

To verify whether GhBLH7-D06 can target the promoter of GhPAL-A06 and regulate its expression, the expression vectors pGreenII62-SK-GhBLH7-d06 and pGreenII62-SK-GhOFP3-D13 were constructed by double luciferase experiment, and the report vector pGreenII0800-LUC-pGhPAL-A06 containing GhPAL-A06 promoter was constructed. The ratios of LUC to REN (LUC/REN) in tobacco leaves were determined by *A. tumefaciens* injection and pGreenII62-SK empty vector was used as control. The results show that LUC/REN ratio in pGreenII62-SK-GhBLH7-D06 group was significantly lower than that of control group, while there was no significant difference for the LUC/REN ratio between pGreenII62-SK-GhOFP3-D13 group and the control group, which also indicated that GhBLH7-D06 could directly target binding to the promoter of GhPAL-A06 to inhibit its expression and ultimately inhibit the lignin biosynthesis, and GhOFP3-D13 might participate in the inhibition function of GhBLH7-D06 as a cofactor (Figure 6a,b).

For the Y1H experiment, we cloned the 1500-bp upstream ATG sequence of *GhPAL-A06* as its promoter sequence. After sequencing, we constructed the Y1H vector pHIS2-pGhPAL-A06 and the AD expression vector pGADT7-GhBLH7-D06. The yeast strain Y187 was co-transfected with pHIS2-pGhPAL-A06 and pGADT7-GhBLH7-D06. After the positive clones were verified by PCR, the yeast plaque growth of the experimental group and the control group was observed on the screening medium supplemented with 3-AT. It was found that the experimental group (pHIS2-pGhPAL-A06 + pGADT7-GhBLH7-D06) with added 150 mM 3-AT can grow normally on SD-Trp/-Leu/-His screening medium but cannot grow normally in empty vector control group, which also indicated that GhBLH7-D06 can regulate the expression of *GhPAL-A06* by directly binding to the promoter sequence of *GhPAL-A06* (Figure 6c).

## 3. Discussion

TALE superfamily transcription factors are a class of proteins with the special homeodomain, which are atypical DNA binding domains composed of 63 amino acids (aa). Compared with the traditional homeobox domains, they insert three additional aa residues (P-Y-P) between the first and second helices [46]. According to the differences of other domains, TALE superfamily transcription factors can be divided into two families, BEL1-Like and KNOX, which always function as heterodimers that are structurally and functionally related [46]. According to different functions, BEL1-Like transcription factors are often divided into five groups: tuberization and root growth, leaf morphology, OFP partners, meristem function, and ovule morphology. The functions of members of the same type are often overlapping and redundant [45,47]. In *A. thaliana*, BLH7 transcription factors belong to the group of OFP partners. In the same classification, BLH6 homologous proteins are mainly involved in the synthesis and regulation of plant SCW, especially the lignin-rich intercellular fiber cell wall [40], while there are few reports on the homologous genes of *BLH5*, *BLH7*, and *BLH11*.

In the previous study, we cloned and analyzed the function of the *BLH6* homologous gene *GhBLH6-A13* in regulating plant SCW, especially lignin biosynthesis. Similar to *A. thaliana*, GhBLH6-A13 can interact with GhKNAT7, but GhBLH7-D06 cannot interact with GhKNAT7 [45]. By analyzing the expression pattern of BLH7 transcription factor in cotton, it was found that *GhBLH7-D06* was dominant in the secondary wall thickening stage of stem and cotton fiber development, indicating that GhBLH7-D06 may also play an important role in stem development or morphogenesis and cotton fiber SCW biosynthesis. The induced expression pattern showed that the expression level of *GhBLH7-D06* was significantly decreased under the infection of *V. dahliae*. Knockdown of the expression of *GhBLH7-D06* resulted in higher Verticillium wilt resistance in silenced cotton plants, indicating that it is a negative regulator of Verticillium wilt resistance. BEL1-Like transcription factor regulates lignin synthesis by repressing the expression of genes in phenylpropanoid metabolic pathway, thus affecting the cell wall composition of vascular tissue and participating in the regulation of cotton Verticillium wilt resistance. Interestingly, GhBLH7-D06 transcription factor has similar functions with other reported homeobox transcription factors such as HDTF1 and GhHB12, both of which show negative regulation of Verticillium wilt resistance [19,20].

**Figure 7:**
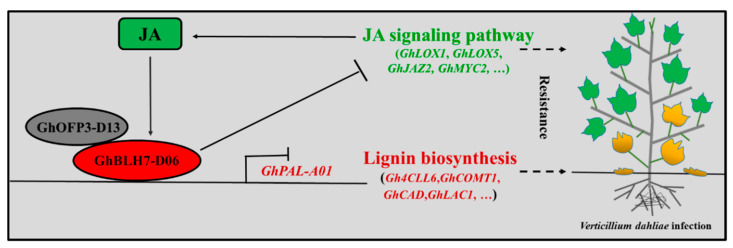
A schematic model of BEL1-Like protein GhBLH7-D06 participate in the negative regulation of cotton Verticillium wilt resistance.

The expression pattern analysis of *GhBLH7-D06* showed that it was predominantly expressed in root, stem, petal, and the secondary wall thickening stage of fiber development. In addition, *GhBLH7-D06* was induced by MeJA and GA, which are representative phytohormones regulating plant resistance and development, respectively [48]. In the regulation of Verticillium wilt resistance, the cross action of phytohormones and the hierarchical response between phytohormones and downstream metabolites are very common phenomena [26,49]. We speculate that the transcription factor GhBLH7-D06 is involved in JA signal pathway of Verticillium wilt resistance regulation and GA signal pathway of cotton fiber development regulation. Of course, we need more experimental evidence to verify the latter. Moreover, GhBLH7-D06 could interact with GhOFP3-D13 which could be induced by SA and respond to the treatment of *V. dahliae*, indicating that the complex formed by GhBLH7-D06 and GhOFP3-D13 may be a bridge for the interaction between JA and SA. In general, GhBLH7-D06, as a negative regulator, is not only involved in the regulation of cotton Verticillium wilt resistance by inhibiting the expression of genes related to lignin biosynthesis, but also forms a negative feedback inhibition process with phytohormone JA; that is, its expression can be induced by JA and it can inhibit the expression of both JA synthesis and signal transduction-related genes to participate in JA response to *V. dahliae* infection (Figure 7).

In addition to the interaction between BEL1-Like transcription factors and KNOX proteins to form heterodimers, they can also form complexes with OFP transcription factors and participate in the regulation of plant growth and development [50,51,52,53,54,55]. Due to the conservation of the binding domain, their members interact more frequently, but few of their functions have been identified. In the regulation of plant growth and development, the reported functional members have high homology, for example KNAT7–BLH–OFP complex in model plant *A. thaliana*, GhKNL1 interactions with GhOFP1 and GhOFP4 in cotton, and GhBLH7-D06/GhOFP3-D13 complex in this study [50,56]. We hope that more interaction modes can be identified and their functions can be revealed. The transcriptional regulation mode of protein complexes on downstream genes is less reported, and there are few studies on the targeted binding sites of the protein complexes. This is more restrictive for the in-depth analysis of the regulatory mechanism of TALE/OFP complex and the construction of related regulatory networks. We need to conduct more in-depth research in this field.

## 4. Materials and Methods

### 4.1. Plant Materials, Growth Conditions and Stress Treatments

Upland cotton (*Gossypium hirsutum* L. cv. “TM-1”) which was grown at Anyang (AY), Henan, China, was used for gene cloning and the tissue/organ quantitative real-time RT-PCR analysis. The roots, stems, leaves, flower, ovules, and fibers of “TM-1” were collected to analyze GhBLH7-D06 expression of different cotton tissues.

Upland cotton “TM-1” and tobacco (*Nicotiana benthamiana*) seedlings were grown in soil-filled pots under greenhouse conditions of 25/22 °C (day/night) with a 16-h light/8-h dark cycle. Leaves from four-week-old “TM-1” plants were used to investigate *GhBLH7-D06* expression changes under different treatments. Hormone treatments were performed by spraying the plants with 5 mM IAA, 0.5 mM GA, 100 mM MeJA, 1 mM SA, or double-distilled water as control. IAA, GA, MeJA, and SA were dissolved in water. Plant–pathogen interaction analyses were performed by spraying spore suspensions of *V. dahliae* strain “*Vd991-GFP*” dipping the roots into a “*Vd991-GFP*” conidia suspension (2 × 10^5^ conidia per mL). Water was used as a control treatment [8].

### 4.2. Gene Cloning, Multiple-Sequence Alignment and Phylogenetic Analysis

To amplify the CDS and promoter of *GhBLH7-D06* (*Gh_D06G0030*), we designed primers using Oligo 7. The full-length CDS and promoter fragment of *GhBLH7-D06* was cloned from cDNA and DNA of TM-1 root. The fragments were inserted into pBI121 vector and transformed into *Escherichia coli* competent cells (*E. coli* DH5a) for sequencing. Multiple sequence alignment was conducted using ClustalX software (ver. 1.83). The phylogenetic tree was constructed using the neighbor-joining method implemented in the Molecular Evolutionary Genetics Analysis (MEGA) software 7.1 with a bootstrap value of 1000. The primers used for gene cloning are listed in Table S2.

### 4.3. Subcellular Localization

Prediction of GhBLH7-D06 protein subcellular localization was performed by WoLF PSORT (http://www.genscript.com/wolf-psort.html). In addition, the coding region of *GhBLH7-D06* without the termination codon was cloned into the pBI121-GFP vector to construct plasmid pBI121-GhBLH7-D06-GFP driven by the 35S promoter. Both the recombinant plasmid pBI121-GhBLH7-D06-GFP and empty vector pBI121-GFP wrapped with gold powder were transferred into onion epidermal cells cultivated on MS plates using a desk type particle gun PDS-1000/He system (Bio-Rad, Hercules, CA, USA) with the parameters: particle bombarding running distance of 9 cm, rupture disk pressure of 1300 psi, and vacuum degree of 28 mmHg. The onion tissues after bombardment were transferred onto new MS agar medium incubating at 25 °C for 12 h in dark, and the green fluorescence of the cells was observed using a fluorescence microscope (Leica DM2500, Solms, Germany).

### 4.4. Expression Analysis

To analyze gene expression levels, total RNA was purified using the RNAprep Pure Plant Kit (TIANGEN, Beijing, China) according to the manufacturer’s instructions. First strand synthesis of cDNA was performed using ReverTra Ace qPCR RT Kit (Toyobo, Osaka, Japan) according to the manufacturer’s instructions. The qRT-PCR was performed on an Mastercycler ep realplex Real Time PCR system (Eppendorf AG, Hamburg, Germany) by using the fluorescent intercalating dye SYBR Green in the detection system. A cotton polyubiquitin gene (*GhHis3*, GenBank accession No. AF024716) was used as a standard control in the RT-PCR and the relative changes were calculated with 2-DCt [57]. The primers used for the PCR amplification are listed in Table S2.

### 4.5. Virus-Induced Gene Silencing (VIGS) of GhBLH7-D06

A 300-bp fragment from the ORF (open reading frame) of *GhBLH7-D06* was inserted into the TRV:*00* plasmid, and it was digested with the restriction enzymes *EcoRI* and *BamHI* to generate the TRV:*GhBLH7-D06* construct. TRV1, TRV:*GhBLH7-D06,* and TRV:*00* were then introduced into *Agrobacterium* strain GV3101. *Agrobacterium* strains containing TRV1 and *Agrobacterium* strains containing TRV:*GhBLH7-D06* or TRV:*00* were mixed in equal amounts and infiltrated into the cotyledons of 10-day-old “TM-1” seedlings by syringe infiltration to generate the control (TRV:*00*) and *GhBLH7-D06*-silenced (TRV:*GhBLH7-D06*) cotton. TRV:*CHLI* (*Magnesium-chelatase subunit I*) was used as a positive control as previously described [58]. Because the etiolated leaf phenotype appeared in the TRV:*CHLI* plants at 10 days after infiltration, we selected that as the time to inoculate the seedlings with *Vd991-GFP* and to take samples for calculating the silencing efficiency. The primers used for PCR amplification and vector construction are listed in Table S2.

### 4.6. Fungal Pathogen Cultivation and Inoculation

*V. dahliae* strain *Vd991-GFP* was incubated on potato dextrose agar (PDA) for 1 week and then inoculated into Czapek broth on a shaker at 120 rpm at 25 °C for 3–4 days until the concentration of spores reached 10^8^ spores/mL [59]. *V. dahliae* infection assays were performed by root dipping with spore suspension (2 × 10^5^ spores mL^−1^), while control plants were mock-treated with distilled water at the same time [8]. The disease index was scored by using at least 20 plants per treatment and repeated at least three times according to Xu et al. [8]. For fungal colonization analysis, longitudinal sections of epicotyls were dissected at 2 weeks after inoculation and photographed under a fluorescence microscope (Leica DM2500, Solms, Germany).

### 4.7. Histochemical Test

Hand-cut cross-sections of epicotyl samples were taken at 4 dpi from 10 inoculated and 10 mock-treated plants of each group. For histochemical analysis, 2-cm-long segments were excised and preserved in a mixture of acetic acid: formalin: ethanol (5:5:90, *v:v:v*). The distribution of lignin was examined using Wiesner reagent staining [60]. The cross sections were incubated for 10 min in a phloroglucinol solution (2% in 95% ethanol) or 95% ethanol (staining control), then treated with 18% HCl for 5 min, and directly observed under bright-field conditions with a fluorescence microscope (Leica DM2500, Solms, Germany).

### 4.8. Yeast Two-Hybrid and Library Screening Assay

The cDNA library of stem and leaf bud for Y2H screening was constructed with The Matchmaker Gold Yeast Two-Hybrid System (Clontech, Mountain View, CA, USA). The cotton GhBLH7-D06 gene was fused with the GAL4 DNA-binding domain in pGBKT7 to ensure that there was no autoactivation and toxicity caused by the X-a-Gal (5-bromo-4-chloro-3-indolyl-a-D-galactopyranoside) assay in yeast; then, the GhBLH7-D06 fusion protein was used as bait to identify the interacting proteins. To detect the protein–protein interactions between GhBLH7-D06 and the identified proteins, the full-length GhOFP3-D13 was cloned into pGADT7. The bait and prey plasmids were subsequently co-transformed into Y2H Gold cells. The transformed yeast cells were then cultured and detected on DDO (SD-Trp/-Leu) or QDO (SD-Trp/-Leu/-His/-Ade + X-α-gal) media that contained 20 µg/mL X-α-Gal. The detailed protocol followed that of the Matchmaker^TM^ Gold Yeast Two-Hybrid System (Clontech, Mountain View, CA, USA).

### 4.9. BiFC Assay

To generate the BiFC constructs, the full-length of *GhBLH7-D06* and *GhOFP3-D13* were cloned and inserted into linearized pSPYNE or pSPYCE vectors which were digested with *XmaI and SacI* using an infusion enzyme to obtain the GhBLH7-D06-nYFP and GhOFP3-D13-cYFP. The empty vector used as negative controls for the BiFC assays. All vectors were transformed into *N. benthamiana* plants via the *A. tumefaciens* strain GV3101. Fluorescence signals in leaf epidermal cells were observed using a confocal microscope (Olympus FV1200, Tokyo, Japan). The primers used for vector construction are listed in Table S2.

### 4.10. Dual-Luciferase Reporter Gene Assay

The 1500-bp promoter sequence of *GhPAL-A06* were amplified and cloned into the reporter vector pGreenII0800-LUC to generate LUC-pGhPAL-A06. The full-length *GhBLH7-D06* and *GhOFP3-D13* were amplified and cloned into the effector vector pGreenII62-SK to generate SK-GhBLH7-D06 and SK-GhOFP3-D13, respectively. Vectors were transformed into *A. tumefaciens* strain GV3101 and used to infiltrate young *N. benthamiana* leaves for transient expression. After 2 days of infiltration, firefly luciferase and *Renilla spp.* luciferase activities were measured using dual-luciferase reporter assay reagents (Promega, Madison, WI, USA) with a Multimode Plate Reader (Perkin-Elmer, Waltham, MA, USA). The primers used in the dual-luciferase reporter assays are listed in Table S2.

### 4.11. Yeast One-Hybrid Assay

The coding sequence of *GhBLH7-D06* was cloned into a pGADT7 vector at the *EcoRI* and *BamHI* sites to create pGADT7-GhBLH7-D06 prey plasmids. A 1500-bp full length *GhPAL-A06* promoter sequence was cloned into a pHIS2 vector to generate bait carrier. The pGADT7-GhBLH7-D06 construct and the bait carrier were subsequently co-transformed into Y187 yeast cells. The transformed yeast cells were grown and detected on SD-Trp/-Leu (DDO) and SD-Trp/-Leu/-His (TDO) media that were supplemented with 150 mM 3-amino-1,2,4-triazole (3-AT) (TDO + 150 mM 3-AT) to evaluate protein-DNA interactions based on growth ability. The primers used in the dual-luciferase reporter assays are listed in Table S2.

## 5. Conclusions

In this study, we identified a cotton homeodomain transcription factor, GhBLH7-D06. This transcription factor is expressed in secondary wall thickening stage of cotton fiber and vascular tissues (root and stem), and it could be induced by MeJA and respond to *V. dahliae* infection. Silencing the *GhBLH7-D06* gene enhanced the resistance to *V. dahliae* infection and increased lignin synthesis and JA signal transduction compared with the control plants in cotton. Besides, An OFP family protein GhOFP3-D13, which is almost root specific, can interact with GhBLH7-D06 and negatively responds to *V. dahliae* infection. These results indicate that the GhBLH7-D06/GhOFP3-D13 complex can negatively regulate resistance to Verticillium wilt of cotton by inhibiting lignin biosynthesis and JA signaling pathway.

## Supplementary Materials

Supplementary materials can be found at https://www.mdpi.com/1422-0067/21/19/7126/s1.

## Abbreviations

TALE	Three-amino-acid-loop-extension
SCW	Secondary cell wall
VIGS	Virus-induced gene silencing
Y2H	Yeast two-hybrid
BiFC	Bimolecular fluorescence complementary
OFP	Ovate family protein
Y1H	Yeast one-hybrid
IAA	Auxin
SA	Salicylic acid
GA	Gibberellin acid
JA	Jasmonic acid
MeJA	Methyl jasmonate
ROS	Reactive oxygen species
TF	Transcription factor
ABA	Abscisic acid
BR	Brassinosteroid
CK	Cytokinin
AA	Amino acid
